# A mixed-methods evaluation of a longitudinal primary–secondary school transitions support intervention

**DOI:** 10.3389/fpsyg.2024.1252851

**Published:** 2024-10-10

**Authors:** Charlotte Louise Bagnall, Elizabeth Stevenson, Darel Cookson, Frederick Jones, Nicholas James Garnett

**Affiliations:** ^1^Manchester’s Institute of Education, The University of Manchester, Manchester, United Kingdom; ^2^School of Psychology, Sandwell Council, Sandwell, United Kingdom; ^3^Nottingham Trent University, Nottingham, United Kingdom

**Keywords:** school transitions, emotional wellbeing, intervention, longitudinal, skills-based curriculum

## Abstract

**Introduction:**

Primary–secondary school transitions are critical transitions for children that can be emotionally demanding longitudinal experiences, which can positively and negatively impact future emotional wellbeing and mental health. However, interventions that have been developed to reduce the negative outcomes children commonly experience are limited in number, sustainability, and reach and rely on a cross-sectional approach, as opposed to longitudinal evaluations. The current study evaluates *Transitions 5–7*, a universal, class-based 9-week intervention to develop children’s awareness and ability to cope with the multiple changes experienced over primary–secondary school transitions.

**Methods:**

The evaluation utilized a mixed-methods approach, combining both quantitative outcome and qualitative process intervention evaluation. For the outcome evaluation, a quasi-experimental research design was used, and children of the intervention and comparison groups completed a questionnaire in Year 5 (*n* = 185), Year 6 (*n* = 217), and Year 7 (*n* = 162), which assessed their self-reported perception of Transitions Worries, Transitions Excitement, Emotional Wellbeing, Parental Support, and Coping Efficacy. To understand the implementation of *Transitions 5–7*, three focus groups were conducted with Year 6 children, 3 interviews with teachers, and 1 interview with the Transitions Manager of the local government education authority during the project, who developed *Transitions 5–7*.

**Results:**

The outcome evaluation found that children participating in the intervention showed a decrease in Transitions Worries and an increase in Transitions Excitement and Coping Efficacy compared with the comparison group, resulting in a lowered impact on Emotional Wellbeing over time. The need for a more systemic approach to primary–secondary school support provision, which is gradual, has a distinct delivery and follows a skills-based curriculum, was discussed in the process evaluation. Meta-inferences drawn demonstrate the importance of gradual emotional centered transitions provision embedded within Years 5, 6, and 7.

**Discussion:**

The present study makes a unique empirical contribution in demonstrating the need and viability to take a preventative as opposed to a curative approach to primary–secondary school transitions support provision and begin early in Year 5. Conceptual and methodological implications for future research and implications for educational policy and practice are discussed.

## Introduction

To date, intervention research on primary–secondary school transitions is limited by (1) not holistically evaluating the impact on children (e.g., research has looked at ‘transitions-specific concepts’ such as social and academic adjustment with a lack of focus on children’s emotional wellbeing), (2) intervention design (e.g., there is a lack of early-intervention support, with no programs beginning in Year 5), and (3) weak evaluation (e.g., there is no intervention evaluations which have measured outcomes at the individual level across three school years [beginning in Year 5], using a mixed-methods approach). Furthermore, as discussed below, to date, a deficit-orientated discourse pervades primary–secondary school transitions research and practice, which extends to the instruments used within research studies. The present study evaluates *Transitions 5–7*, a skill-based, early-intervention approach program, using a mixed-methods approach over 3 school years. Overcoming the above limitations, this study provides a novel empirical insight into the development of children’s transitions experiences and emotional wellbeing over primary–secondary school transitions longitudinally from both qualitative and quantitative perspectives. In addition, this research provides conceptual innovation through assessing changes in children’s transitions worries and excitement separately, as distinct constructs, in uniquely predicting change in emotional wellbeing over time.

## Background

### Significance of primary–secondary school transitions

In countries such as England, Scotland, Wales, and Northern Ireland, the majority of children move to secondary school at age 11 years. Although a normative part of life, transitioning from primary to secondary school, is a critical developmental period, which is believed to have positive and negative impacts on emotional wellbeing and mental health (White, 2020). During this time, children negotiate multiple, simultaneous changes in identity (primary/secondary school child, child/young person), school environment, friendship groups, teaching styles, and academic expectations (Bagnall, 2020). Primary–secondary school transitions also occurs at a time when children are experiencing hormonal changes associated with puberty, in addition to school-based pressures, such as academic national Standard Assessment Tests in England, which can further impact children’s cognitive and social processing (Ng-Knight et al., 2019) and perpetuate feelings of instability and anxiety accompanying the change of school in this stage of development (Bagnall et al., 2020; Bharara, 2020). For example, although many children feel optimistic about the opportunities primary–secondary school transitions afford, a substantive body of research also shows that adjusting to school transitions changes can be difficult (Demkowicz et al., 2023; Garner and Bagnall, 2024), and navigating school transitions unsuccessfully can have ongoing short- and long-term wide-ranging social, academic, and emotional implications (White, 2020; Donaldson et al., 2023).

There is a growing body of research with a clear shared contemporary conceptualization of transitions as a multi-dimensional ongoing process which spans across multiple domains and contexts (Jindal-Snape, 2016). This emerging clear and consistent conceptualization within the field is having, and will continue to have, significant advantageous implications for research designs, study findings and their interpretation, especially in informing policy and practice (Hannah et al., 2023). This has been recognized within several landmark international systematic literature reviews, which have been published in the past 5 years, to rigorously and systematically synthesize international primary–secondary school transitions research published since 2008 (Jindal-Snape et al., 2021; Bagnall and Jindal-Snape, 2023; Hannah et al., 2023). Our conceptualization of primary–secondary school transitions also aligns with Jindal-Snape (2016) *Multiple and Multi-dimensional Transitions Theory (MMT)*, which conceptualizes the changes children negotiate over primary–secondary school transitions, as concurrent “transitions,” which occur in multiple domains (e.g., social, academic) across multiple contexts (e.g., school, home) and are multi-layered. For example, alongside the child, the child’s peers and parents will also be negotiating multiple transitions in the same and different domains, which may interact with or instigate other transitions for the child (which will be discussed in further detail below).

Negotiating multiple transitions simultaneously can impact children’s ability to cope as outlined in Coleman’s (1989) *Focal Theory of Change*, which posits that sequential rather than simultaneous change can be easier for children to cope with and have psychosocial and emotional consequences. Theoretically this can be extended with Baumeister et al.’s (2007) *Depleted-Resource Hypothesis* which outlines that over time, frequent concurrent stressors can significantly draw on self-regulatory capacities and disrupt cognitive processing. The negative impact of navigating cumulative change has been shown empirically in the context of primary–secondary school transitions in the UK; a longitudinal study by Rice et al. (2011) found that the number and not severity of school concerns over primary–secondary school transitions to predict peer problems, generalized anxiety, and depression.

At face value, there appears to be considerable school transitions research. Often, research is limited by not holistically considering the impact of primary–secondary school transitions on the child, as most research neglects children’s emotional wellbeing (Jindal-Snape et al., 2020; White, 2020). Instead, most research focuses on the social and academic implications of primary–secondary school transitions, despite emotional wellbeing shown to be directly linked with children’s academic functioning (Vassilopoulos et al., 2018) and social adjustment (Coffey, 2013). For example, in line with broader developmental cascade studies (Petersen et al., 2022), children who experience poor emotional wellbeing have an increased risk of educational disruption, poor academic attainment, and social maladjustment. Thus, supporting children’s emotional wellbeing over primary–secondary school transitions is a significant primary concern, to support cognitive and social processing (Ng-Knight et al., 2019).

As a result, there is limited understanding of (a) how primary–secondary school transitions might impact children’s emotional wellbeing and (b) the trajectory of change in children’s emotional wellbeing during this time (Bagnall and Jindal-Snape, 2023). For example, despite the positive correlation between wellbeing and criteria of positive transitions experiences, such as school bonding and behavioral adjustment, there is still no model befitting the relationship between school transitions and wellbeing (Bharara, 2020). The present study aimed to address this gap by (1) examining the trajectory of change in children’s transitions appraisals (specifically worry and excitement) over primary–secondary school transitions in predicting emotional wellbeing over time and (2) whether early-intervention transitions provision could predict positive outcomes within this model (e.g., reduce Transitions Worries and increase Transitions Excitement, improving children’s emotional wellbeing over time).

### Primary–secondary school transitions intervention research

As outlined above, primary–secondary school transitions can be emotionally demanding, and longitudinal critical periods for children and significant others within their ecosystem. Therefore, there is a need for greater focus on gradually supporting children’s emotional wellbeing over primary–secondary school transitions, taking an early-intervention approach. This is recognized in research (Beatson et al. 2023) by those supporting children during transitions periods (e.g., parents/guardians, teachers) (Bagnall et al., 2020) and also within policy (Department for Education, 2021), especially when considering the recovery of children’s emotional wellbeing following the COVID-19 pandemic (Bagnall et al., 2022). Given the under-resourced nature of schools when addressing long-standing mental health concerns, preventative emotional wellbeing support provision may now be preferred (Department for Education, 2023).

Recognizing primary–secondary school transitions as a key entrance point for school-based intervention support, a number of intervention programs have been developed to improve children’s experiences during this time. This has been shown in a systematic literature review by Beatson et al. (2023), which synthesized and evaluated available evidence to date, pertaining to the design, selection, implementation, and evaluation of primary–secondary transitions interventions, to identify which interventions are most efficacious, feasible to deliver, and suitable for universal and targeted populations within schools. In doing so, the review identified 26 interventions which focused on supporting social–emotional (i.e., peer-relationships, self-concept, and mental health) and educational (i.e., school engagement, academic achievement) outcomes. Moreover, a handful of intervention evaluations assessed the impact of intervention support on mental health outcomes, such as depression, anxiety, and emotional symptoms; although it is worth noting that all focused on symptomology as an outcome, as opposed to improving mental health literacy.

Furthermore, there were limited interventions which focused on supporting children’s emotional wellbeing over primary–secondary school transitions, with most interventions centered around the practicalities of school transitions, preparing children for the new ways of learning, and social changes (Jindal-Snape et al., 2020; White, 2020). All of these programs additionally take a curative, as opposed to a preventative approach to transitions support provision. The present intervention overcomes these limitations by introducing transitions provision in the Spring term of Year 5 (second to last year in primary school in England) and continuing to the final term of Year 6 (last year of primary school in England), which is discussed further below in the *“Longitudinal design and evaluation”* section.

Furthermore, Beatson et al. (2023) systematic literature review found that intervention research lacks (a) rigorous evaluation methods and (b) consistent measurement of outcomes, which are discussed below.

### Rigorous evaluation methods

#### Longitudinal design and evaluation

As discussed above, there is a lack of longitudinal primary–secondary school transitions research, and this methodological limitation extends to intervention research. For example, intervention research is limited in the (a) length of intervention follow-up evaluations, with only one study examining outcomes across three school years (Jindal-Snape and Cantali, 2019); (b) the precision within longitudinal evaluations (specifically the timing and level of comparisons drawn) as this one study made group-level comparisons as opposed to individual-level comparisons (Jindal-Snape and Cantali, 2019), and (c) the timing of baseline assessments (specifically there has been no transitions research which has collected data in Year 5 in primary school in the UK, which is important to obtain a true baseline assessment, as recommended in previous research) (Bagnall, 2020). Furthermore, to date, primary–secondary school transitions research has relied on mostly cross-sectional designs, consisting of isolated, one-off assessments of outcomes just before or after the ‘move’ to secondary school, e.g., pre/post (often with no or little baseline assessments) (Rice et al., 2011), as opposed to longitudinal research studies, measuring outcomes over multiple time points (West et al., 2010).

In part, this methodological limitation of relying on short-term evaluation studies may be due to primary–secondary school transitions being conceptualized as an ‘event’ when it is an ongoing process of assimilation and adaptation that occurs over several years. This has resulted in uncertainty within the field pertaining to the length of time that it takes for children to emotionally adjust to school transitions (Jindal-Snape and Cantali, 2019) and a partial picture of the optimal point to begin intervening to develop children’s transitions awareness, knowledge, and skills to prepare them for primary–secondary school transitions. This could have led to an incomplete understanding from research studies that have informed policies and practices. For instance, if transitions are understood to take place in the break between primary and secondary school, it could lead to transitions support only being offered immediately before (e.g., in the preceding term) and/or after transitioning to secondary school (e.g., in the following term).

Thus, by recognizing primary–secondary school transitions as an ongoing process, and the dynamic nature of emotional wellbeing in the context of primary–secondary school transitions, this study overcomes these longitudinal design and evaluation methodological limitations to date, as *Transitions 5–7* begins in the Spring term of Year 5. For the evaluation of *Transitions 5–7*, the current research will also use a longitudinal approach across three school years (Years 5 and 6 in primary school and Year 7 in secondary school).

#### Mixed-methods evaluation

Despite a preponderance of evidence supporting the benefits of conducting mixed-methods evaluations of interventions (Moseholm and Fetters, 2017), there are very few mixed-methods interventions used within primary–secondary school transitions research, in spite of the recommendations (Jindal-Snape and Cantali, 2019). This is problematic, as to understand the context and variability in implementation, but also to ensure that the program has ecological fit, social validity, and can be sustained over time to work at scale, combining both qualitative process and quantitative outcome intervention evaluation, is paramount (Durlak and DuPre, 2008; Fohlin et al., 2021). Therefore, the current research will use an integrated mixed-methods design, allowing for holistic and credible meta-inferences to be made. To do this, the first-hand perspectives of children and practitioners are drawn on to provide context-specific evidence, pertaining to the sustainability and practical utility of intervention support (Demkowicz et al., 2023).

### Consistent measurement of outcomes

It is common for primary–secondary school transitions interventions to vary with regards to using differing designs (top-down vs. bottom-up approach), inclusion criteria (universal, targeted, and proportionate universalism), and their breadth of foci (e.g. social-emotional wellbeing, teaching and learning, social adjustment), which can impact replication across different education contexts. For further discussion of the range of approaches taken to improve student transitions, see Bagnall (2020) and Symonds et al. (2023). Thus, to date, primary–secondary school transitions interventions lack consistency in the outcomes they target and, specifically, the psychometrics used, which means that we have a partial picture of which interventions are most efficacious, feasible to deliver, and suitable for universal and targeted populations within schools. Below, we outline the outcomes, as well as the psychometrics that are used in the present research, and rationale for doing so.

### Coping efficacy

Coping efficacy is a core self-evaluation mechanism, pertaining to one’s belief in being able to manage the demands of a perceived contextual barrier and the emotions aroused to interpret challenges in an enabling, as opposed to a debilitating way (Sandler et al., 2000; St Clair-Thompson et al., 2017). Coping efficacy is thus conceptually different from adversity concepts commonly investigated within transitions research (Bagnall and Jindal-Snape, 2023), such as resilience (which focusses on normative function that results in good outcomes in spite of serious threats to the individuals wellbeing) (Masten, 2001) and buoyancy (the ability to successfully deal with typical setbacks and challenges) (Martin and Marsh, 2008), in that it focusses on children’s “perceived ability”. Furthermore, it is worth noting that these adversity constructs are vaguely defined and conceptually weak (Hart and Heaver, 2013). Therefore, rather than being viewed as prescriptive constructs, resilience and buoyancy should be viewed more as holistic and dynamic emergent concepts, which deserve further research.

Previous research has shown that children who have positive expectations prior to primary–secondary school transitions (Waters et al., 2014) and exhibit greater emotional self-efficacy (Nowland and Qualter, 2020) are more likely to positively adjust following transitions. Shedding further light on these findings in the context of coping efficacy, qualitative retrospective case study research has shown that children who discussed having belief in their ability to cope with the changes associated with school transitions prior to transitioning schools (especially if they had experienced previous transitions) discussed finding school transitions easier (Bagnall et al., 2021b). However, while intervention research has shown preliminary evidence of variability in children’s coping efficacy when in primary school (Bagnall et al., 2021a), a gap exists in the literature in understanding the longitudinal trajectory of coping efficacy in predicting emotional wellbeing in secondary school.

Thus, there is a need to (a) measure children’s perceptions of their coping efficacy over time and (b) provide equitable opportunities for all children to develop coping efficacy leading up to primary–secondary school transitions. Narrowing empirical gaps (a) and (b), the present research explores the extent to which coping efficacy scores are improved following participation in *Transitions 5–7* (see overview below). *Transitions 5–7* is a skill-based drama intervention, focused on nurturing children’s coping efficacy through developing transitions knowledge and applying learnt transitions strategies.

#### Parental support

As outlined in *MMT* theory, primary–secondary school transitions are multi-dimensional, where transitions for an individual child can trigger unintentional transitions for others within their ecosystem and vice versa, leading to an almost ongoing ripple effect across the ecosystem (Jindal-Snape, 2016). This theory is important when considering the role of parental support, which can provide a crucial source of continuity for children when relationships with classmates and teachers can be unstable (Bagnall et al., 2020) and can help to mitigate academic, social, and emotional maladjustment during this time (White, 2020), especially for children who are more emotionally vulnerable to poorer transitions experiences (Bagnall et al., 2021c). However, within school transitions intervention research, parent support is understudied (Jindal-Snape and Cantali, 2019; Beatson et al., 2023). Thus, the present study also assessed children’s perceptions of parental support to measure the trajectory of these relationships over primary–secondary school transitions.

#### Transitions worries and excitement

Although many children feel optimistic and excited about the opportunities primary–secondary school transitions affords, a negative, deficit-orientated discourse pervades primary–secondary school transitions literature (Jindal-Snape et al., 2021), with a substantive body of research conceptualizing school transitions as a difficult, worrying time, challenging children’s coping abilities and negatively impacting their emotional wellbeing (McCoy et al., 2020). This negative discourse extends to the psychometric instruments used to assess children’s transitions experiences and/or emotional wellbeing, which commonly feature a deficit-oriented discourse from the names of instruments to the wording of items and response format (Bagnall and Jindal-Snape, 2023). This deficit orientation can be leading (i.e., it encourages respondents to understand their experiences in a negative fashion), impacting the validity of the findings, in addition to possibly giving a negative message to children about the impact of primary–secondary transitions, e.g., “worrying about stopping the bad’ in comparison to ‘creating excitement about the good’.

In addition, there is a dearth of standardized, robust and accessible quantitative instruments to sensitively assess children’s emotional wellbeing in the context of primary–secondary school. This is due to both conceptualization and instrumentation limitations, as shown in a systematic review by Bagnall and Jindal-Snape (2023) on child self-report instruments used to assess children’s emotional wellbeing and primary–secondary school transitions experiences. The study found only 27% of papers measured both transitions experiences and emotional wellbeing, and instead researchers to date are inadequately using measures which assess [a] solely primary–secondary school transitions experiences, and [b] using several scales within a single study to measure emotional wellbeing (as no measure fully captures emotional wellbeing over primary-secondary school transitions) and not measuring primary-secondary school transitions. Bagnall and Jindal-Snape (2023) speculated that this may have been influenced by author’s conceptualisation of transitions in terms of change, move, or a time point, and as a result of this, they may have operationalized primary–secondary school transitions in terms of a static time point and measured changes in emotional wellbeing pre- and post-transitions to secondary school. This is inhibiting progress within the field, as these designs are unable to holistically assess children’s emotional wellbeing *in the context of* primary–secondary school transitions as a process of adaptation over time.

Thus, the present research will strengthen the methodological and conceptual foundations that underpin understanding of primary–secondary school transitions within the field by (a) adapting an existing instrument to include additional items to assess children’s appraisals of school transitions, specifically related to *the context of* primary–secondary school transitions in the UK and (b) measuring transitions worries and transitions excitement separately as distinct constructs. Both (a) and (b) will make a first step in overcoming poor psychometric properties shown in instruments within the field and provide a novel paradigm shift in measuring whether transitions worries and transitions excitement can uniquely predict change in children’s emotional wellbeing over time (Jindal-Snape and Cantali, 2019). This will enable us to make the first step in examining whether changes in emotional wellbeing are reflective of primary–secondary school transitions worries and excitement and context driven or other environmental or personal factors (e.g., parent support and/or coping efficacy).

### Rationale

Primary–secondary school transitions can be emotionally demanding (positive and negative) experiences for children. There is a need for preventative, early-intervention support leading up to this period, which is in line with previous research (Bagnall et al., 2021a) and theory (Coleman, 1989; Baumeister et al., 2007; Jindal-Snape and Bagnall, 2023). The present research will make the first step in doing this by evaluating the efficacy of *Transitions 5–7* (discussed below), in supporting children’s emotional wellbeing over primary–secondary school transitions. To do this, *Transitions 5–7* focuses on increasing children’s transitions excitement and coping efficacy and reducing transitions worries, which is in line with the key tenants of the *MMT* theory (Jindal-Snape, 2016). In keeping with the conceptualization of transitions as an ongoing dynamic process, a longitudinal design was used to evaluate the efficacy of *Transitions 5–7* across 3 school years, following a mixed-methods interventional evaluation approach, combining both qualitative process and quantitative outcome intervention evaluation.

#### Design

The present research used a quasi-experimental mixed methods design. This included a pre-, post-, and delayed post-follow-up survey design, with a parallel process evaluation, which is outlined below.

As outlined by Hannah et al. (2023), transparency, consistency and congruence between philosophical perspectives, conceptualisations, theoretical frameworks, and methodology is paramount to determine the robustness of a study and interpretations drawn yet has been limited in transitions research studies to date. This is vital to advance primary–secondary school transitions research, policy, and practice (Jindal-Snape et al., 2021). Thus, the outcome and process evaluation were undertaken from a critical realist perspective, acknowledging the existence of a reality that has regularities and is contextually influenced, which was tested by the research hypotheses and questions. This is congruent with our conceptualizations and theorization of emotional wellbeing and primary–secondary school transitions discussed above within the introduction section.

#### *Transitions 5–7* intervention

*Transitions 5–7* is a 9-week universal emotional-centered transitions intervention, which starts in the spring term of Year 5, to develop children’s awareness and ability to cope with the multiple changes they will experience over primary–secondary school transitions. The content and structure of *Transitions 5–7* was designed by the Transitions Manager of the local government education authority (N.B. the Transitions Manager has since moved jobs) and drew on her 18 years of first-hand experience supporting primary–secondary school transitions, in addition to previous research, including Bagnall’s (2020) *Talking about School Transitions* intervention. The program consists of nine lessons, of which lessons one to five were delivered by the Transitions Manager and lessons six to nine were delivered by the class teacher. This enabled comparison in how the quality of implementation of *Transitions 5-7*, differed based on who delivered the sessions, which was assessed within the intervention process evaluation. Each of the nine lessons (see Table 1) lasted approximately one hour, which is considered an optimal length for children of this developmental age (Merrell and Gueldner, 2010), and consisted of a variety of individual, group, and class-based drama activities, which aimed to improve children’s spoken and written emotional expression in preparation for primary-secondary school transitions.

**Table 1:** Timetable of *Transitions 5–7* and summary of each lesson.

Date	Half Term	Theme	Aim
8th–12th March 2021	Spring (Y5)	Lesson 1: Asking for help	Helping pupils develop the skill of asking for help when it is needed, especially from people (adults and children) that they may not know or trust yet.
24th–28th May 2021	Summer 1 (Y5)	Lesson 2: Identifying independence	Helping pupils recognize the times they are/know how to be independent. This independence looks very different between primary and secondary school.
12th July–16th July 2021	Summer 2 (Y5)	Lesson 3: Relationships with new staff	Not only how to build relationships but how to ‘recognize and know your audience’ and how to be the appropriate version of themselves.
27th Sept–1st Oct 2021	Autumn 1 (Y6)	Lesson 4: Choices	Support pupils to identify the important factors when deciding which secondary school to apply for.
22nd–26th Nov 2021	Autumn 2 (Y6)	Lesson 5: Expectations	This will look at the pupil’s expectations of secondary school, managing expectations and dispelling the myths!
24th–28th Jan 2022	Winter (Y6)	Lesson 6: Navigating	This will look at navigating around new surroundings, including understanding timetables and systems.
14th–18th March 2022	Spring (Y6)	Lesson 7: Making friends	Focusing on how to make a friend. This is a skill that many pupils have never really had to do.
9th–13th May 2022	Summer 1 (Y6)	Lesson 8: Structure/life changes	How life at secondary school will be different, including new routines before and after school.
11th–15th July 2022	Summer 2 (Y6)	Lesson 9: So, what now?	The final session will look at what the pupils are excited about and looking forward to, in addition to any concerns the pupils may have and how to resolve these for them.

Recognizing that primary–secondary school transitions are a sensitive period for children, comparison group schools were asked to carry out transitions provision as they usually would in the Local Authority. This included support at the end of Year 6 (following National Assessments), such as class discussions about moving to secondary school, Year 6 parents’ information evenings about primary–secondary school transitions, visit days, and exchange of secondary school information.

## Quantitative outcome evaluation

### Outcome evaluation hypotheses

The theoretical constructs operationalized in the evaluation of *Transitions 5–7* were Emotional Wellbeing, Transitions Worries, Transitions Excitement, Coping Efficacy, and Parental Support. These variables were assessed across six time points: Time 0 (Summer Year 5), Time 1 (Autumn Year 6), Time 2 (Winter Year 6), Time 3 (Summer Year 6), Time 4 (Autumn Year 7), and Time 5 (Winter Year 7); however, due to attrition, complete data were only available at three time points. This was acceptable as these time points occurred during Years 5, 6, and 7, providing data across three school years (henceforth: Times 0, 1, and 2). To examine whether *Transitions 5–7* is more effective over time, versus concentrated, and whether outcomes are stable, it was hypothesized that:

Hypothesis 1: Transitions Worries will have a reduced negative impact on children’s Emotional Wellbeing over time (from Year 5 to Year 7). This negative impact will be greater for the comparison group than the intervention group.

Hypothesis 2: Transitions Excitement will have a positive impact on children’s Emotional Wellbeing over time (from Year 5 to Year 7). This positive impact will be greater for the intervention group than the comparison condition.

Hypothesis 3: Coping Efficacy will have a positive impact on children’s Emotional Wellbeing over time (from Year 5 to Year 7). This positive impact will be greater for the intervention group than the comparison condition.

Hypothesis 4: Parental Support will be positively predictive of Emotional Wellbeing regardless of condition or time point.

### Method

#### Outcome evaluation

##### Participants

Table 2 shows a breakdown of participant numbers by gender and group (intervention vs. comparison) over time. All schools within a local authority of the West Midlands in the United Kingdom were invited to participate in the *Transitions 5–7* intervention evaluation, and schools were matched across conditions based on sociodemographic and academic performance characteristics by the Transitions Manager. It is worth noting that all schools that participated in the present intervention evaluation had above the national average percentages of children in receipt of pupil premium funding (a government grant given to schools in England to support disadvantaged pupils from low socioeconomic backgrounds) and were located in areas of significant social and economic deprivation.

**Table 2:** Participant numbers in each condition and across time.

Time and group	Female (*N*)	Male (*N*)	Prefer not to say (*N*)	Total (*N*)
**Time 0 (Year 5)**				
Intervention	59	59	1	119
Comparison	38	23	5	66
Total	97	82	6	185
**Time 1 (Year 6)**				
Intervention	58	47	2	107
Comparison	57	47	6	110
Total	115	94	8	217
**Time 2 (Year 7)**				
Intervention	56	42	2	100
Comparison	36	22	4	62
Total	92	64	6	162

##### Instruments

The main body of the questionnaire (see Appendix 1) consisted of four scales. For constructs emotional wellbeing, coping efficacy and parent support, published instruments were used and are presented in Table 3, whereas Transitions appraisals (Transitions worries and Transitions excitement) were measured using an adapted instrument, which is discussed below.

**Table 3:** Each construct, corresponding measure, N of items, sample item and response format.

Construct	Measure	*N* of items	Sample item	Response format
Emotional wellbeing	*Warwick-Edinburgh Mental Wellbeing Scale* (Anthony et al., 2022)	7 items	I’ve been feeling optimistic about the future (item 1)	5-point Likert Scale (1: none of the time, 2: rarely, 3: some of the time, 4: often, 5: all of the time)
Coping efficacy	*Coping Efficacy Scale* (Sandler et al., 2000)	7 items and an additional 4 items were included which made the scale more relevant to school transitions. These items pertained to how likely the child thinks they will be able to seek support from a: classmate, parent/carer, primary school and secondary school teacher when problems come up in the future?	Overall, how satisfied are you with the way you handled your problems during the last month? Would you say…? (item 1)	4-point Likert scale (1: Not at all satisfied, 2: A little satisfied, 3: Pretty well satisfied, 4: Very satisfied)
Parent support	*Child and Adolescent Social Support Scale, Level 1 (CASSS)* (Malecki et al., 2000)	9 items	Express pride in me (item 1)	3-point rating scale (2: yes, 1: sometimes, 0: not true).

#### Transitions appraisals (transitions worries and transitions excitement)

Recognizing the limitations within school transitions research instruments to date, to assess children’s negative (worries) and positive (excitement) appraisals of transitions, in the present research, the items used by Smith et al. (2008) were adapted. The items are organized around relevant domains of primary to secondary school transitions (academic/social/organizational) but do not balance these for pupil’s potential excitement and concerns within these domains as it was not a focus of the quantitative element of their research. The seven items relating to the helpfulness of others were removed as we utilized specific instruments to measure this. The original survey used ‘I am worried about…’ as a survey stem, of which four items were retained, and “I am looking forward to…” as a survey stem, of which six items were retained. We rephrased the latter to ‘I am excited about’, in place of ‘I am looking forward to’ to ease readability and avoid adaptations across time points (e.g., in secondary school), as the same stem could be used in each school setting. Three new ‘worried about’ items were added, which specifically related to the context of primary–secondary school transitions in the UK, drawing on previous qualitative research (Bagnall et al., 2020); this included item 5: ‘I worry about academic pressure’; item 8: ‘I worry about safety at school’; and item 10: ‘I worry about new rules’. Items were rated using the four-point Likert scale (1, strongly disagree, 2: disagree, 3: agree, and 4: strongly agree). However, Transitions Worries and Transitions Excitement were assessed as distinct constructs and scored separately.

#### Procedure

The studies involving humans were approved by Keele University Psychology Faculty Research Ethics Committee (PS-210176). The studies were conducted in accordance with the local legislation and institutional requirements. Written informed consent for participation in this study was provided by the participants’ legal guardians/next of kin. Data collection, using online surveys, was completed by children in class time. Before beginning the survey, all children read the same information sheet by their class teacher and gave written assent. To generate anonymous identifiers to match respondents over time, children created a five-item secret code, which consisted of three items according to Respondent Generated Personal Code items by Ripper et al. (2017), which have been shown to generate a percentage match of 99.7%, in addition to the child’s birth month and gender. The same procedure was replicated at each time point. Following data collection, the children were debriefed, offered the opportunity to ask questions, and pointed to sources of support, and the research aims were explained.

### Results

#### Outcome evaluation

##### Data preparation

Unfortunately, there were missing data at time points of 1, 3, and 5 for the comparison group, meaning these time points had to be excluded from the analyses, as there was not sufficient comparison data available to be able to impute missing data. However, there were data from each of the three school years—Time 0 (Spring Year 5), Time 2 (Autumn Year 6), and Time 4 (Autumn Year 7). We decided to collapse down the time points to a year level, providing an appropriate spread of time points across primary–secondary transitions; Time 0 is pre-intervention, Time 2 at the end of primary school, and Time 4 post-transitions. For clarity, these will be addressed as Time 0, 1, and 2 within the analyses below. Within these data, there were some systemically missing data (i.e., schools had not completed data collection at certain time points) and/or data missing at random (i.e., individual participants not completing or partially completing individual instruments). There were 15 (3.3%) fully complete cases across all time points (please see Supplementary material); however, there were sufficient data at each time point to utilize imputation (Zhang, 2016). Imputed data were only utilized for mixed models. As the instruments had a variety of outcomes, all data were log transformed (Gelman and Hill, 2006). Within the condition variable, the intervention was dummy coded as positive.

##### Data analysis

The internal reliability of the psychometric instruments was assessed using greatest lower bound (glb) (Peters, 2014; McNeish, 2017). This was computed for each instrument at each time point (see Table 4).

**Table 4:** Instrument reliability evaluation—greatest lower bound (95% confidence intervals).

Instruments	Time 0	Time 1	Time 2
Emotional Wellbeing	0.64 (0.60, 0.76)	0.79 (0.75, 0.87)	0.79 (0.73, 0.87)
Transitions Worries	0.90 (0.88, 0.94)	0.74 (0.69, 0.81)	0.74 (0.68, 0.81)
Transitions Excitement	0.79 (0.72, 0.86)	0.70 (0.62, 0.76)	0.62 (0.52, 0.75)
Coping Efficacy	0.83 (0.80, 0.89)	0.91 (0.89, 0.94)	0.90 (0.88, 0.94)
Parental Support	0.81 (0.78, 0.88)	0.90 (0.88, 0.93)	0.93 (0.90, 0.96)

The results in Table 4 suggest that the instruments have acceptable internal consistency; however, it is worth noting that *Warwick-Edinburgh Mental Wellbeing Scale* at Time 0 and Transitions Excitement at Time 2 is on the low side even though it is acceptable.

##### Bayesian mixed effect models

Data were analyzed using a series of Bayesian mixed-effect models. Models were specified using the brms R package (Bürkner, 2018), and 5,000 iterations were fitted with weakly informative priors over four chains. Models specified with a Student distribution as model fit was superior to categorical and normal distributions, with a Student distribution providing robustness toward skew and kurtosis (see Supplementary material). Model convergence was assessed with plot trace and density graphs, Rhat, effective sample sizes, and posterior probability checks within brms (Bürkner, 2017; Nalborczyk et al., 2019). The models were built from a null model (intercept only) and assessed model fit using out-of-sample predictions estimated using leave-one-out cross-validation (Vehtari et al., 2017). For the assessment of models’ predictive performance, we used expected log predictive density (ELPD).

In outputs below, estimates in which the lower and upper credible intervals do not contain zero (i.e., one can be confident they are statistically meaningful) are marked with a double asterisk (**), and estimates that do contain zero but the error is greater than the difference from zero to relevant upper or lower credible interval (i.e., one can assume it could be statistically meaningful but with caution) are marked with a single asterisk (*).

#### Quantitative evaluation

This model addresses all the outcome evaluation hypotheses mentioned in the rationale section. To estimate emotional wellbeing over time, and whether this was dependent on condition (intervention or comparison) and other factors such as transitions excitement or worry, parental support, and coping efficacy, they were modeled as fixed effects. As data were nested—occasion measurement grouped within individual and individuals grouped within schools—this was recognized in the model. Therefore, the model was fitted with random intercepts and random slopes for the effect of time on both classrooms and pupils nested within classroom. We explored the most appropriate method to model time in the same fashion, arriving at a second-order polynomial (combined linear and quadratic function). The ELPD difference between the null and final models was ELPD -357.5 (SE = 34.1) (for full breakdown of models and ELPD please see Supplementary material).

The model was coded thus:


$$\begin{array}{l} EMW\sim \text{poly}{\left(\text{Time},2\right)}^{*}\;{\text{Intervention}}^{*}\;\left(PS+CE+TW+TE\right)+\\ \left(\text{poly}\left(\text{Time},1\right)|s|\text{ID}:\text{School}\right)+\left(\text{poly}\left(\text{Time},1\right)|p|\text{School}\right) \end{array}$$

N.B. EMW = Emotional Wellbeing, poly(Time,2) = the “Time” factor as a second order polynomial, PS = Parental support, CE = Coping efficacy, TW = Transitions worries, TE = Transitions excitement, poly(Time,1) = the “Time” factor as a first order polynomial.

Outputs from Model 1 are shown in Table 5 above.

**Table 5:** Results of quantitative evaluation—Bayesian mixed model outputs.

Parameter	Est.	Est. error	Low 95% CI	High 95% CI	
Time 1	−5.32	4.31	−13.83	3.05	*
Time 2	−1.82	3.47	−8.68	5.16	
Intervention	0.15	0.14	−0.12	0.43	*
Parental Support	0.24	0.09	0.06	0.41	**
Coping Efficacy	0.56	0.07	0.43	0.70	**
Transitions Worries	−0.05	0.06	−0.16	0.06	*
Transitions Excitement	0.05	0.07	−0.08	0.19	
Time 1 * Intervention	2.44	5.35	−7.97	12.89	
Time 2 * Intervention	−3.75	4.60	−7.97	5.50	
Time 1 * Intervention * Parental Support	−1.55	4.65	−10.72	7.52	
Time 2 * Intervention * Parental Support	3.71	3.90	−4.03	11.34	
Time 1 * Intervention * Coping Efficacy	3.23	3.37	−3.34	9.78	*
Time 2 * Intervention * Coping Efficacy	1.02	3.27	−5.31	7.43	
Time 1 * Intervention * Transitions Worries	−8.11	3.11	−14.22	−1.96	**
Time 2 * Intervention * Transitions Worries	−5.37	2.99	−11.33	0.40	*
Time 1 * Intervention * Transitions Excitement	4.28	3.60	−2.83	11.13	*
Time 2 * Intervention * Transitions Excitement	4.61	3.04	−1.39	10.55	*

N.B. – estimates in which the lower and upper credible intervals do not contain zero (i.e., one can be confident they are statistically meaningful) are marked with a double asterisk (**), and estimates that do contain zero but the error is greater than the difference from zero to relevant upper or lower credible interval (i.e., one can assume it could be statistically meaningful, but should interpret with caution) are marked with a single asterisk (*).

From these results, we concluded the following in relation to the hypotheses:

In line with hypothesis 1, children participating in the intervention had a statistically meaningful decrease in Transitions Worries compared with the comparison group, resulting in a lowered impact on Emotional Wellbeing. This was sustained over time, as children participating in the intervention group had a sustained statistically meaningful decrease in Transitions Worries and subsequent positive impact on Emotional Wellbeing from Time 0 to Time 1 (Year 5 to Year 6) and from Time 0 to Time 2 (Year 5 to Year 7) compared with the comparison group.

In line with hypothesis 2, children participating in the intervention had a statistically meaningful increase in Transitions Excitement compared with the comparison group, resulting in an increased impact on Emotional Wellbeing. This was sustained over time, as children participating in the intervention had a sustained statistically meaningful increase in Transitions Excitement and subsequent positive impact on Emotional Wellbeing from Time 0 to Time 1 (Year 5 to Year 6) and from Time 0 to Time 2 (Year 5 to Year 7) compared with the comparison group.

In line with hypothesis 3, children participating in the intervention had a statistically meaningful increase in Coping Efficacy and subsequent positive impact on Emotional Wellbeing compared with the comparison group. However, for children participating in the intervention, there was a statistically meaningful increase in Coping Efficacy from Time 0 to Time 1 (Year 5 to Year 6) but not from Time 0 to Time 2 (Year 5 to Year 7) and subsequent positive impact on Emotional Wellbeing compared with the comparison group.

In line with hypothesis 4, parent support was a statistically meaningful predictor of Emotional Wellbeing regardless of condition or time point. This is borne out as Parental Support is not a statistically meaningful predictor in the elements of the model, which evaluates the intervention (the interaction terms).

## Qualitative process evaluation

### Process evaluation research questions

To obtain detailed insight in identifying components of *Transitions 5–7* that were most critical in generating outcomes, the intervention process evaluation addressed the following research questions:

1. How has *Transitions 5–7* been implemented within schools, and what factors affect implementation?

This included the exploration of:

- The perceived understanding and usefulness of *Transitions 5–7*?
- The quality of *Transitions 5–7*, specifically teacher responsiveness and child engagement and whether this differed dependent on who delivered the program?
- Any barriers and facilitators which affected the successful implementation of *Transitions 5–7* and recommendations for future practice?

### Method

#### Participants

All participants within the process evaluation were drawn from the same schools as the outcome evaluation and recruited via opportunity sampling. Twenty-four Year 6 (aged 10 and 11 years) children (14 male and 12 female) from three of the intervention primary schools participated in the focus groups. Three teachers (all female), from three of the intervention schools, participated in an interview, as did the Transitions Manager at the time of the project (who developed *Transitions 5–7* and is referred to within the analysis as “Programme Developer”). All participants self-selected to participate in the process evaluation. Our sample size and number/composition of groups are considered moderate to identify 90% of the themes and theoretical data saturation for a given topic (Guest et al., 2017); this was decided given that the area of interest is specific and thus would reasonably lead to rich, focused discussion, alongside our focus on sample diversity (Nyumba et al., 2018).

#### Materials

Focus group and interview semi-structured questions were developed to guide the discussions (see Appendix 2–4), which included prompt and follow-up questions where necessary.

#### Procedure

##### Child focus groups

The studies involving humans were approved by Keele University Psychology Faculty Research Ethics Committee (PS-210176). The studies were conducted in accordance with the local legislation and institutional requirements. Written informed consent for participation in this study was provided by the participants’ legal guardians. Before data collection, children read an information sheet and were asked to adhere to key ground rules, and informed assent was obtained. Once the allotted time ended (30 min), the children were thanked and debriefed, offered the opportunity to ask questions and pointed to sources of support for them to access should they require it.

##### Adult interviews

All teachers from the participating intervention schools were invited to participate in an interview. Self-selected participants who indicated interest were then emailed an information sheet and consent form containing details regarding the interview, and a convenient time and date were arranged. Following data collection, participants were thanked, debriefed, offered the opportunity to ask questions, and pointed to sources of support for them to access they should require it.

#### Data analysis

To protect participants’ identity, audio-recordings and transcripts were anonymized at source and stored on password-protected computers. Audio-recordings were then transcribed using verbatim transcription. As the intent of the analysis was to describe, summarize, and interpret surface-level patterns in semantic content from the sample, data were analyzed using Hybrid Thematic Analysis within a contextualist framework. Hybrid Thematic Analysis combines a blended inductive, data-driven process and deductive, empirically and theory-driven process to interpret raw data (Fereday and Muir-Cochrane, 2006; Xu and Zammit, 2020). This approach was considered appropriate for the present research, as it allowed for the similarity and difference between pre-existing understanding within the field to be identified, in addition to the generation of new insights from the data.

Three authors (CB, DC, and FJ) followed Braun and Clarke’s (2019) six stages of Thematic Analysis which is presented in Table 6 with the thematic maps created shown in Table 7.

**Table 6:** Reflexive thematic analysis steps.

Process of reflexive thematic analysis	
Familiarization with data set	Each interview was transcribed verbatim and then read to ensure credibility. Anonymity was ensured at this stage as children received a number and any identifying names were removed. (CB, DC,FJ)
Generating Initial Inductive codes	FJ identified features of the data that were considered pertinent to the research question. This stage was conducted blind, as FJ had not seen the coding framework, which CB developed *a priori* based on the research question
Generating Initial deductive codes	DC conducted deductive coding, applying the coding framework to the data to identify meaningful units. DC also noted new units of meaning that were not detailed by the preliminary codes, within the coding framework. This served as a data management tool for organizing segments of similar or related data and provided a clear trail of evidence, for the credibility of the study
Search for themes	CB then scrutinized the data, sorted and organized inductive and deductive codes across the transcript groups, clustered under themes directly relating to the research questions. Inductive codes assigned to segments of data were either separate from the predetermined codes or they expanded a code from the coding framework.
Reviewing themes	Themes’ external homogeneity and internal homogeneity were then reviewed and refined by CB, DC, and FJ to ensure that they were accurate and valid representations of the data, exhibiting clear and identifiable distinctions between teachers and children, but also cohered meaningfully.
Defining and naming themes	Theme and sub-theme names and definitions were refined through discussion between CB, DC and FJ and thematic maps were created.

**Table 7:** A thematic table to show themes and subthemes.

1. Systemic support and accountability	2. Distinctive delivery of transitions provision	3. Gradual transitions support	4. Skill focused transitions curriculum
1a. School-based practice	2a. Not who, but how?	3a. Exposure and “the goldilocks zone”	4a. Developing transitions awareness
1b. Policy change	2b. Formation of safe space	3b. Timing matters	4b. Managing expectations
	2c. Guidance	3c. Continued support	4c. Translating skills
			4d. Emotional development

### Results

Four main themes, namely, 1*. Systemic support and accountability, 2. Distinctive delivery of transitions provision, 3. Gradual transitions support*, and 4. *Skill-focused transitions curriculum* were identified across the interviews and focus groups. As shown in Table 7, each theme has corresponding sub-themes, which are explored separately below using illustrative quotes from participants.

#### 1 Systemic support and accountability

Accountability within transitions provision was consistently discussed within the adult interviews, as paramount to facilitate change in how primary–secondary school transitions preparations were approached: “it was a hard job because nobody had done it before, there was lots of things bringing schools together, so lots of hearts and minds but there was no accountability for anybody doing anything with me” (Program Developer). It was acknowledged that there is a need for accountability within transitions provision, facilitated by change at a school-level, through change in *1a. School-based practice* and *1b. Policy change.*

##### 1a School-based practice

At the school-level, bringing schools together to form a transitions community, with a shared transitions strategy, was especially important in establishing *Transitions 5–7*: “looking at system wide approaches that we could do, with the understanding that every school would still do the right thing by their community, what was needed was a strategy involved across all of our schools” (Program Developer). Integral to this was buy-in from school senior leadership teams, and the local authority, having one central school-transitions manager: “Just that kind of management system meant that everything was just better prepared, because the person doing it was outside of any connection to any individual trust or school, everybody had got that buy-in and so having somebody who manages it and in charge of transitions it is crucial” (Program Developer), and embedding the program into PSHE: “the way we planned it, it was evidencing PSHE” (Teacher 2).

##### 1b Policy change

The program developer discussed induction and transitions being two very different entities, and the latter, transitions, requiring gradual, long-term support provision, which needs to be embedded within school-based practice: “there’s a difference between transitions and induction. And I think most schools do induction well, they do not do transitions particularly well because it is longer term than end of SATS and a few weeks in” (Program Developer). For this to happen more widely, it was acknowledged that transitions provision needs to be embedded into the curriculum at a more systemic level: “I would really like to see some sort of curriculum expectations in Key Stage 2, focused on developing skills, and having a skills-based curriculum, so our actual skills, our personal skills, not just our geographical and our scientific skills” (Program Developer). This would also avoid overlap with topics covered within the curriculum: “we overlapped with PSHE and then we overlapped with the transitions” (Teacher 3).

#### 2 Distinctive delivery of transitions provision

*Transitions 5–7* was distinctive in *how* transitions provision was delivered, and *who* delivered the program was discussed as secondary to this, as captured in the subtheme *(3a) Not who but how?* Inherent in how transitions provision was delivered, was the need for *(3b) Formation of a safe space* and the importance of *(3c) Guidance* to enable teachers to feel confident in delivering *Transitions 5–7*.

##### 2a Not who but how?

On one hand, an external facilitator delivering *Transitions 5–7* helped to bring a novel perspective, which was special for the children: “having somebody come in and deliver the lessons was something special for the children, I think they enjoyed it with someone coming in” (Teacher 2) and gave a palpable credibility to these sessions, as she was perceived to be an expert in transitions: “I think [program developer] should teach us the lessons because she’s experienced it a lot more than our class teachers have” (Focus group 2). However, it was clear that the value of these sessions was not dependent on who was delivering them, instead *how* it was delivered, which was different from children’s usual lessons, in that children were encouraged to test out their learning through drama: ‘I like how we acted it out instead of just like speaking on what to do, we actually acted it out as if we were there’ (Focus group 1). Inherent in this novel perspective, and style of teaching was new expectations, to foster independence: “I had to be very clear with the children by saying I’m not marking anything. Nothing you do is for me. Absolutely everything you do in these sessions is for you. It’s all just for your own experience” (Program Developer).

##### 2b Formation of a safe space

Providing children with a safe space to talk and ask questions about secondary school was integral to *Transitions 5–7* in supporting openness, transparency, and emphasis of ‘no wrong answers’ which encouraged relationship building and trust: ‘because of my openness and my transparency with the children, we got some really good relationships going’ (Program Developer). This openness and transparency allowed the children to feel safe and ask questions freely: “the lessons formed a safe place where you can ask questions” (Focus group 2). Children also discussed already having a rapport and level of comfort in discussing secondary school with their class teacher: “I thought it was good doing the split because we had [program developer] who helped us like act everything out and then we had [teacher] who like spoke to us and we could speak like more freely than we could with [program developer]” (Focus group 1).

##### 2c Guidance

Successful delivery of *Transitions 5–7* was dependent on forward-planning, which was prescriptive in terms of when in Year 5 and 6 transitions lessons are delivered but gave school flexibility to select the week, day, and time: “having the pre-planned timetable worked well, I’d set aside weeks, in my diary for the whole two years sessions, so I was never scratting for time” (Program Developer). Teachers were also given the program developer’s lesson plans, which some teachers found helpful as they were: “No, I think the lesson plans that were sent through that I was delivering were really comprehensive, they were easy to follow, resources were there” (Teacher 1), but others felt more information was needed, especially about the underpinnings of the program: “I found it quite difficult following someone else’s planning, there wasn’t really much information on the planning” (Teacher 2) and “the lesson plans could be combined into a booklet, that could be sent out to schools, and you could see…the lessons from start to finish” (Teacher 3). Some teachers also felt that worksheets could be helpful: *“*the children having some sort of reminder written of what they have done in each lesson, so they could look back on it” (Teacher 2).

#### 3 Gradual transitions support

Taking a gradual, progressive approach, to transitions support, provision was paramount as discussed in sub-theme *3a. Exposure and the goldilocks zone.* Integral to this was the early-onset approach that *Transitions 5–7* embodied in beginning transitions provision in Year 5 (unique to typical provision currently employed within the field), as discussed in sub-theme *3b. Timing matters*, and *3c. Continued support* into secondary school being gold-standard.

##### 3a Exposure and the “goldilocks zone”

Transitions provision was deemed best when at the “goldilocks zone” in terms of the pace of sessions being gradual and spread out to provide exposure but avoid overwhelm: “I think the timing works. I think the way we spread it out, we try to do one a half term which works, it wasn’t too overpowering for the children, it wasn’t too much or too little” (Program Developer). This early exposure was also discussed as important in preparing children for induction days: “We started it in Year 5 because then we could use it into Year 6 and at our induction days but if we started it straightaway in Year 6, then we would not be as prepared for our induction days as we were” (Focus group 1).

##### 3b Timing matters

There was shared agreement by all stakeholders that beginning transitions support provision in Year 5 was more helpful than delaying this provision until Year 6, as the gradual approach facilitated greater opportunities for the children to (a) practice skills: “in Year 5, the transitions lessons were getting you prepared by practicing things for when it comes” (Focus group 2); (b) ask questions: “the children were more confident as well to come and ask me questions and ask me things if they needed to” (Teacher interview 2); (c) discuss their feelings: “You sit, and you think back over the lessons, and I think that they are now very used to talking about emotions” (Teacher 1); and (d) reflect: “I feel…starting it in Year 5 gave them longer to think about it” (Teacher interview 1).

##### 3c Continued support

To compliment, the transitions support in school, transferring learning to settings outside school, was discussed as important: “Yes, because she gave us some tasks outside of the lessons as well, like to do like outside of school” (Focus group 1), particularly to the home, but there was acknowledgement that parents would need support for this: “I think a parent session would be useful, and to give parents the tools as well to talk about it and to not put their worries onto their child would be useful’ (Teacher 1).

Continuing transitions provision into secondary school was discussed as gold standard to support children over primary–secondary school transitions: “So, you know, best case scenario there is time spent at primary school developing these skills and then time spent at secondary school homing in on those skills” (Program Developer), especially in terms of keeping emotional centered discussions going: “that there’s some merit in them being able to talk about how they are adapting because it is a big difference from primary” (Teacher 1).

#### 4 Skill-focused transitions curriculum

*Transitions 5–7* followed a skill-focused transitions curriculum, which focused on *4a. Developing transitions awareness*, and *4b. Managing expectations*, then *4c. Translating skills* into practice through habit formation and ‘testing out’ capabilities. Together, this supported children’s *4d. Emotional development.*

##### 4a Developing transitions awareness

*Transitions 5–7* helped to develop children’s awareness towards primary–secondary school transitions changes: “So, at first, I felt quite anxious about what was going to happen and then I felt fine because at least I know what’s going to happen and everyone’s probably going to be in the same boat as me” (Focus group 3). However, there were clear individual differences in terms of when children had begun thinking about secondary school, which meant that for some children, the first transitions session was their first experience of thinking about their next chapter: “in Year 5 before the lesson, I did not really think much about secondary school and I did not think about like how it would change from primary school and then when I got the lessons, I then thought more about how secondary school would be different from primary school” (Focus group 1). However, for other children, the first *Transitions 5–7* lesson coincided with when they started developing worries toward secondary school. This meant for some children, the usefulness of beginning transitions support in Year 5 was not realized until Year 6 when they understood their next chapter more through exposure in the lessons: “Basically, at first, I thought it was unhelpful in Year 5 because I did not really have a clue about secondary school but then when we went to Year 6, yeah, it became more helpful” (Focus group 3), but for other children, the utility was immediate.

##### 4b Managing expectations

Managing children’s expectations was at the forefront of how *Transitions 5–7* was delivered: “at first when I found out I was going to a different school, I wasn’t really too sure what to expect, but doing those lessons, it helped me prepare for it and I know what’s coming, so I can be a bit more confident” (Focus group 3). This transparency also permeated through the openness in which secondary school was discussed: “I think she came in to give us a warning about secondary school, to prepare us for what’s to come, so then we know what to do. I thought that were helpful because before I had the lessons I did not know what to expect” (Focus group 1).

##### 4c Translating skills

From the outset, it was made clear to the children that transitions challenges could not be removed, instead children needed to develop the skills and ‘habits’ to cope and practice them: “These are mindset changes that need to come from the children, these are skill changes that need to come and habits do not change overnight and there needs to be that long term preparation for them, and actually, then you are not even forming the habit until they get to secondary school” (Program Developer). It was quickly made clear that while children were aware of challenges they would experience at secondary school and what they *should* do; children needed opportunities to ‘test-out’ their learning: “It’s not just telling the children what they can do it’s actually preparing them to be able to do that for the first time” (Program Developer), especially using realistic situations: “what was most helpful was giving us realistic situations that will happen in secondary school” (Focus group 3), so children could experience what this would feel like: “I think less talking and more action, like get people to interact with the activities because it’s good to feel it like what you are actually doing, what it will be like at high school” (Focus group 2).

##### 4d Emotional development

*Transitions 5–7* also helped children to understand how they felt towards secondary school and how to manage these emotions: “it helped me see like what emotions I felt about the school, and it made me look at how I can change those emotions to be a better emotion.” (Focus group 1). Developing confidence and self-esteem was discussed as paramount to this: “as prepared as they are, the worries that they have had over the last few years, they need to build that confidence too…because it’s such a big change” (Teacher Interview 1).

## Meta-inferences

To maximize the scientific rigor, efficiency, and yield of mixed-methods research, it is integral that mixing of methods is purposeful in data generation, analysis, and interpretation. However, many mixed-methods interventional evaluation studies fall short in describing the analytical framing and procedures used, in addition to the process of generating and presenting meta-inferences (Fetters and Molina-Azorin, 2020). This may be driven by (a) little acknowledgement of ontological and epistemological positioning within papers, (b) a lack of rubric to follow in the design of mixed-methods research and methodological decision making, and (c) no clear integration framework to draw and present meta-inferences (Tashakkori and Teddlie, 2008), known as the “problem of integration” (Onwuegbuzie and Johnson, 2006, p. 48).

In line with (a), the present study takes a pragmatism-based approach, valuing shared meaning and joint action (Morgan, 2014). Complementing the strengths and weaknesses within both qualitative and quantitative approaches (Shannon-Baker, 2015), and overcoming limitation (b), the present study follows an integrated mixed-methods interventional evaluation design, which recognizes the “equally valued contributions of quantitative and qualitative approaches in the evaluation of complex interventions” (O’Cathain, 2018, p. 17). To do this, the current study utilized a parallel design, where qualitative and quantitative data were first analyzed independently in relation to the research questions they were addressing, to ensure the internal coherence of each strand (Moseholm and Fetters, 2017) and develop a more comprehensive understanding (Demkowicz et al., 2023). Then, building on (c) qualitative and quantitative findings were then merged following a bidirectional simultaneous approach, which is consistent with the study’s use of mixed methods to bring the two strands together to support complementarity and offer greater insights (Fetters and Molina-Azorin, 2020). To do this, a visual merging matrix was created, juxtaposing the findings from the quantitative and qualitative modes of inquiry to draw meta-inferences (Table 8).

**Table 8:** Meta-inferences across the quantitative and qualitative strands of inquiry, drawn from the outcome and process intervention evaluation of *Transitions 5–7*.

Quantitative inferences	Meta-inferences	Qualitative inferences
There was a sustained decrease in Transitions worries and increase in Transitions excitement from Year 5 to Year 7 for children participating in *Transitions 5–7*, which was positively predictive of Emotional Wellbeing.	Meta-Inference One: Transitions support provision needs to take a gradual, progressive approach to best support children’s emotional wellbeing	Transitions provision was deemed best when at the “goldilocks zone” in terms of the pace of sessions being gradual and spread out. This provided time for exposure into what secondary school will be like and for children to understand and manage their emotions, which avoided feelings of overwhelm.
Transitions worries uniquely predicted emotional wellbeing over time. Transitions excitement uniquely predicted emotional wellbeing over time.	Meta-Inference Two: Transitions worries and Transitions excitement are distinct constructs, which children hold different schemas toward	There were individual differences in children’s readiness to discuss primary-secondary school transitions in Year 5, shaped by affective differences in worry and excitement.
Pupils participating in *Transitions 5–7* had a statistically meaningful increase in Coping Efficacy from Year 5 to Year 6 This statistically meaningful increase was not maintained into Year 7 when children were not participating in *Transitions 5–7*	Meta-Inference Three: Skills-based transitions support provision in Year 5 and 6 can scaffold children’s belief in their ability to cope with primary-secondary school transitions	*Transitions 5–7* embodied a novel discourse in making it clear that transitions challenges could not be removed, and instead followed a skills-based curriculum to help children understand, develop and practice transitions skills.

Having outlined inferences that can be drawn from the quantitative outcome and qualitative process evaluation findings, below these findings are integrated to create meta-inferences. As discussed within the introduction section, this approach is consistent with the study’s use of mixed methods to achieve complementarity across strands (Bryman et al., 2008). These meta-inferences represent a more comprehensive and nuanced understanding of the efficacy of *Transitions 5–7* in supporting children’s emotional wellbeing over primary–secondary school transitions. Three meta-inferences were developed:

1. Transitions support provision needs to take a gradual, progressive approach to best support children’s emotional wellbeing.
2. Transitions worries and Transitions excitement are distinct constructs, which children hold different schemas toward.
3. Skill-based transitions support provision in Year 5 and 6 can scaffold children’s belief in their ability to cope with primary–secondary school transitions.

Table 8 illustrates how each of these meta-inferences has been informed and is discussed in further detail within the discussion section.

## Discussion

In sum, despite the importance of supporting children’s emotional wellbeing, and experiences of primary–secondary school transitions, in UK policy (Department of Health and Social Care and Department for Education, 2018; Public Health England, 2019), practice (White, 2020), and research (Bagnall and Jindal-Snape, 2023), the evidence base remains incomplete. To date, the majority of studies, within primary–secondary school transitions research: (a) do not focus on children’s emotional wellbeing (and instead prioritize children’s social or academic adjustment); (b) face methodological design limitations, including lack of baseline assessment and longitudinal follow-up designs with at least three time points; (c) follow a negative discourse, presenting primary–secondary school transitions as an adverse experience; and/or (d) researchers are limited by the lack of a standardized, robust, sensitive, and accessible quantitative instrument to longitudinally assess children’s emotional wellbeing in the context of primary–secondary school transitions. These limitations have resulted in a lack of understanding pertaining to the impact of primary–secondary school transitions on children’s emotional wellbeing and how to improve this period, which will be discussed, in turn, in the context of the present findings.

The current study aimed to evaluate the efficacy of a longitudinal primary–secondary school transitions support intervention, which was designed to develop children’s awareness and ability to cope with the multiple changes experienced over primary–secondary school transitions. Both quantitative and qualitative findings demonstrated that *Transitions 5–7* was effective in doing this by taking a gradual, progressive approach to best support children’s emotional wellbeing, as drawn in meta-inference one. For example, it was found in the outcome evaluation that children in the intervention condition had a sustained increase in Transitions Excitement and decrease in Transitions Worries across time, which was positively predictive of Emotional Wellbeing in Years 6 and 7. These findings have advanced empirical understanding in providing the first evidence that Transitions Worries and Transitions Excitement are separate constructs that both individually predict Emotional Wellbeing over time (discussed further below). In addition, the present findings have made a valuable and original methodological contribution to primary–secondary school transitions research, being the first study to longitudinally assess both children’s emotional wellbeing and their experiences of primary–secondary school transitions at the individual level (as opposed to the group level) over Years 5, 6, and 7. Thus, the present research provides further support for *MMT* theory, recognizing primary–secondary transitions as an ongoing process of adaptation over time (Jindal-Snape, 2016) and overcomes previous cross-sectional and snapshot designs measuring outcomes before and after the transitions to secondary school (Rice et al., 2011).

Extending the outcome evaluation findings pertaining to the empirical value of longitudinal transitions provision in improving outcomes, the process evaluation shed further light on “the how and why” *Transitions 5–7* worked, which has been recommended to advance understanding within the field, and it is important that future intervention research takes a mixed-methods approach, drawing meaningful meta-inferences (Bloyce and Frederickson, 2012; Bagnall et al., 2021a). Practitioners discussed the importance of *Transitions 5–7* sessions beginning early in Year 5 and being spread evenly across Years 5 and 6 to help facilitate a gradual pace of transitions provision. This provided greater opportunities for children to be exposed to school transitions knowledge, practice skills, ask questions, and discuss their feelings, so children feel prepared but not overwhelmed, which has been shown within qualitative research to be a fine balancing act (Bagnall et al., 2020). Recognizing this, reserving PSHE class time during Years 5 and 6 for *Transitions 5–7* sessions was discussed as paramount for the program’s feasibility, as was having an element of flexibility for schools to select the week, day, and time for each session. This is insight that could not be obtained from the outcome evaluation findings alone but nonetheless important to understand the complexity of implementing emotional-centered transitions support during this time and support recommendations for future practice and policy (Education Endowment Foundation, 2019). Thus, the process evaluation findings make a unique and valuable contribution to existing primary–secondary school transitions literature and provides insights that were not available before pertaining to the practical utility of transitions interventions, which have direct practical implications for schools.

Extending Bagnall’s (2020) emotional-centered *TaST* intervention, *Transitions 5–7*, also embodied a novel discourse making it clear that transitions challenges cannot be removed, and instead the importance of developing children’s awareness of what primary–secondary school transitions will be like to manage their expectations. As outlined in meta-inference three, *Transitions 5–7* did this through fostering a skills-based curriculum in Years 5 and 6, to help children to develop new skills and ‘habits’ to adjust to the new school setting. As shown through the process evaluation findings, the transparent skills-based curriculum was shown to help children recognize, understand, and manage their emotions, supporting their emotional development, which is in line with the core components of socioemotional learning programs (Lawson and Parker, 2019), and previous research has shown the importance of managing children’s expectations during this time, to ensure children do not feel falsely prepared (Bagnall et al., 2020).

The efficacy of *Transitions 5–7* in taking a skill-based approach and early-intervention focus on supporting children’s emotional wellbeing was further shown in the outcome evaluation findings, which found Coping Efficacy to have a statistically meaningful impact on Emotional Wellbeing from Year 5 to Year 6 but not Year 6 to Year 7 for intervention group children only. These findings provide support for the efficacy of *Transitions 5–7* in nurturing children’s perceptions of their ability to cope leading up to primary–secondary school transitions. This has implications for the need to begin transitions support in Year 5 to take a universal, early-intervention, and preventative approach in scaffolding children’s coping skills and coping efficacy. This support then needs to be continued to Year 7 through bridging programs, as once the *Transitions 5–7* lessons finished, Coping Efficacy no longer had a statistically meaningful positive impact on Emotional Wellbeing. This is especially important, given that Parental Support was shown to be a statistically meaningful predictor of Emotional Wellbeing regardless of condition and time point. As *Transitions 5–7* did not specifically target Parental Support as a protective factor, these findings suggest that the program may buffer the impact of differences in parent support on children’s emotional wellbeing. However, further research is needed to explore the underlying structure of the relationships between variables (i.e., a nomological network). This will enable researchers to refine the content and delivery of pre-existing emotional-centered primary–secondary school transitions interventions by strengthening the conceptual foundations that underpin primary–secondary school transitions and emotional wellbeing research. This will also enable researchers to identify transferable intervention mechanisms that may hold across different contexts and settings, as recommended as a future direction needed in the field (Donaldson et al., 2023).

The present research has also demonstrated a novel paradigm shift in measuring transitions worries and transitions excitement separately as distinct constructs by trialing some single items specifically related to the context of primary–secondary school transitions in the UK. This draws on recommendations in previous research, pertaining to the negative discourse permeating primary–secondary school transitions (Jindal-Snape et al., 2020), in addition to the limitations in existing instruments which assess children’s experiences of primary–secondary school transitions (Bagnall and Jindal-Snape, 2023). As drawn in meta-inference two, Transitions worries and Transitions excitement were shown to be distinct constructs, which children hold different schemas towards. For example, the outcome evaluation found Transitions Excitement and Transitions Worries to be conceptually distinct constructs, having statistically meaningful distinct impacts on Emotional Wellbeing in Year 6 and Year 7. In other words, children were shown to hold differing schemas, pertaining to what they are looking forward to when moving to secondary school and what they are worried about. The process evaluation data shed further light on these findings, as for some children (particularly those who had not thought about secondary school before the first *Transitions 5–7* lesson or who were more optimistic), the usefulness of *Transitions 5–7* in shaping how they felt toward primary–secondary school transitions and readiness to discuss primary–secondary school transitions was only realized in Year 6 once they had developed greater transitions awareness.

These findings extend previous intervention research findings, especially pertaining to individual differences in children’s feelings towards underestimating and overestimating transitions changes leading up to primary-secondary school transitions (Bagnall et al., 2021a), which, drawing on the present findings, may have unique influences on adjustment. The present research also provides conceptual innovation in demonstrating that Transitions Worries and Transitions Excitement are distinct constructs and need to be both assessed separately when using instruments within the field. This also supports movement within the field for a more balanced discourse pertaining to primary–secondary school transitions (Jindal-Snape et al., 2021) and is in line with *MMT* theory, which recognizes that both appraisals are experienced simultaneously and change over time over primary–secondary school transitions.

However, it could be argued that measuring transitions appraisals (e.g., Transitions Excitement and Transitions Worries) and Emotional Wellbeing separately using different scales is a limitation, as both parameters are shown to vary over time, which is shaped by each other and often detected by very small changes, as shown in the present outcome evaluation findings. Thus, using separate measures could have produced inefficient estimates, especially given that Transitions Excitement has low internal reliability in Year 7 (although not uncommon in primary-secondary school transitions research) (Bagnall and Jindal-Snape, 2023). For example, it could be that the measures used may not be adequately capturing children’s feelings toward the changes they are negotiating in context, and mask-specific adjustment outcomes for different groups of children (especially children who may be underestimating or overestimating transitions appraisals) stunting progress within the field. This is also inconsistent with common metrics agenda concerns, which outlines the significance of a single scale being more sustainable, consistent in terms of usability and greater value for money (Krause et al., 2021). Thus, our findings provide further support for Bagnall and Jindal-Snape (2023) systematic review findings, pertaining to the need for a standardized, robust, sensitive, and accessible quantitative instrument to longitudinally assess children’s emotional wellbeing in the context of primary–secondary school transitions. This is in recognition that conceptually transitions are an adaptation to a change, not the changes in isolation (Jindal-Snape, 2016), and the present findings provide further evidence for the need to conceptualize and operationalize emotional wellbeing in the context of primary–secondary school transitions, as shown through the design and validation of the *Primary-Secondary School Transitions Emotional Wellbeing Scale* by Bagnall et al. (2024).

In sum, the present research has made a unique empirical contribution in demonstrating the need for primary–secondary school transitions provision to take a preventative as opposed to a curative approach and begin early in Year 5. Support for children’s emotional wellbeing should be at the forefront of transitions provision, and this should not end as children leave primary school. To do this, there is a need for a more systemic approach to primary–secondary school transitions provision, with emotional wellbeing central to this, which is in line with educational policy (Public Health England, 2019; Department for Education, 2023). To address this unmet need, there needs to be greater clarity pertaining to the evidence of impact through a novel paradigm shift in conceptualizing and operationalizing emotional wellbeing in the context of primary–secondary school transitions. Integral to this, there is a need for a balanced discourse (considering children’s worries and excitement in the context of primary–secondary school transitions) and a longitudinal approach in future research, which the present research has made the first step in overcoming.

## Limitations

This study has some limitations, one of which is attrition, which meant that 3 as opposed to 6 time points were included within analyses, as there was not sufficient comparison data available. However, despite this, there were data from each of the three school years—Time 0 (Spring Year 5), Time 2 (Autumn Year 6), and Time 5 (Autumn Year 7) with a consistent experimental group. Given the dearth of research with more than two time points, this is still a positive contribution to our understanding of the longitudinal dimension of primary to secondary school transitions. Recognizing this, and as outlined in the result section of the quantitative evaluation, readers are reminded that these data presented should be interpreted with caution; as we recognize in this study, this was an applied study and should be considered as an incremental step in our development of understanding in the field. Another limitation is that fidelity and dosage data could not be reported, as data were only available from 3 of the 5 intervention schools. Finally, the development and evaluation of the *Transitions 5–7* in just one local authority also raises questions about the transferability of the intervention and the generalizability of the results obtained in the present study. Recognizing that this research project presents findings from a pilot evaluation, large-scale randomized control trial research is needed to overcome these limitations.

While we note the limitation of the project occurring within one local authority, this was a major advantage in the delivery of the intervention, in that there was an existing community of practice between the primary and secondary schools. The relationship between primary and secondary school partners is critical in establishing a successful transitions experience for children (Donaldson et al., 2023). Therefore, a wider evaluation is needed to explore the replicability of the findings of the current study in which relationships between primary and secondary schools are less well developed, which the research team are currently implementing.

## Conclusion

The present study makes several original and significant contributions to primary–secondary school transitions research and practice. This the first intervention study to assess children’s transitions appraisals and emotional wellbeing in Year 5 in primary school in the UK and follow children at the individual level, as opposed to the group-level (Jindal-Snape and Cantali, 2019) across three school years, using a mixed-methods approach. Through purposeful integration of equivalently driven mixed-methods in data generation, analysis, and interpretation, the present research contributes to mixed-methods research by demonstrating the use of a clear integrative framework, with a common set of standards, to draw and present meaningful and robust theoretically consistent meta-inferences (Moseholm and Fetters, 2017). Furthermore, this study provides a novel paradigm shift in conceptualizing and operationalizing children’s transitions appraisals in terms of both worry and excitement and finding these constructs to be conceptually distinct, having unique statistically meaningful impacts on Emotional Wellbeing over time. Thus, this study makes the first step in overcoming conceptual and methodological limitations shown in instruments to date within the field, makes empirically informed recommendations for future research in this area, and demonstrates the importance and viability of early intervention in supporting children’s emotional wellbeing over primary–secondary school transitions.

## Data availability statement

The original contributions presented in the study are publicly available. This data can be found at: https://osf.io/4zwrq/?view_only=04ae299871e44b2c94e63ea31d8b493d.

## Ethics statement

The studies involving humans were approved by Keele University Psychology Faculty Research Ethics Committee (PS-210176). The studies were conducted in accordance with the local legislation and institutional requirements. Written informed consent for participation in this study was provided by the participants’ legal guardians/next of kin.

## Author contributions

CB and ES contributed to the conception of the study. CB, ES, and NG contributed to the design of the study. NG performed the quantitative statistical analysis, supported by CB, DC, and FJ. CB developed the meta-inferences and wrote the first draft of the manuscript, and led the qualitative data analysis, supported by FJ and DC. All authors contributed to manuscript revision, read, and approved the submitted version for publication and made a substantial, direct, and intellectual contribution to the study.
