# Supplementary material for: A mixed-methods evaluation of a longitudinal primary–secondary school transitions support intervention
Source: Front Psychol. 2024 Oct 10;15:1252851. doi: 10.3389/fpsyg.2024.1252851 (PMC11500325; doi:10.3389/fpsyg.2024.1252851)
Supplement: Supplementary file 3 [file Data_Sheet_3.PDF]

```
#This code was was written by A RESEARCHER (EMAIL)
#The paper this code relates to is [INSERT PAPER NAME HERE]

#Installs----
#display numbers not exponentials and increase maximum row printing
for summary outputs
remove.packages("brms")
remove.packages("rstan")
if (file.exists(".RData")) file.remove(".RData")

install.packages("StanHeaders", repos = c("https://mc-stan.org/r-
packages/", getOption("repos")))
install.packages("rstan", repos = c("https://mc-stan.org/r-
packages/", getOption("repos")))

options(mc.cores = parallel::detectCores())
rstan_options(auto_write = TRUE)

#Optimise RStan config - Run once!
cat('Sys.setenv(BINPREF = "C:/Rtools/mingw_$(WIN)/bin/")',
    file = file.path(Sys.getenv("HOME"), ".Rprofile"),
    sep = "\n", append = TRUE)

dotR <- file.path(Sys.getenv("HOME"), ".R")
if (!file.exists(dotR))
  dir.create(dotR)
M <- file.path(dotR, "Makevars")
if (!file.exists(M))
  file.create(M)
cat("\nCXXFLAGS=-O3 -Wno-unused-variable -Wno-unused-function",
    file = M, sep = "\n", append = TRUE)

#install packages
install.packages('tidyr')
install.packages('plyr')
install.packages('dplyr')
install.packages('tidyverse')
install.packages('arsenal')
install.packages('ggplot2')
install.packages('psych')
install.packages('brms')
install.packages('mice')
install.packages('DescTools')

#load packages and data####
options(scipen = 999, max.print = 1000000)

library('plyr')
library('tidyr')
library('dplyr')
library('tidyverse')
library('arsenal')
library('ggplot2')
library('psych')
```

```
library('rstan')
library('brms')
library('mice')
library('DescTools')

rstan_options(auto_write = TRUE)

#Load data frame
df = read.csv("wide_T025.csv", na.strings = c("","NA"))

#adjust variable types
df[4:121] = lapply(df[4:121], as.numeric)
df$School = as.factor(df$School)
df$Gender = as.factor(df$Gender)
df$Intervention = as.factor(df$Intervention)

#Generate means for each variable by time point----
df = df %>% mutate(T0_WEMWBS_MEAN =
rowMeans(across(T0_WEMWBS_1:T0_WEMWBS_7), na.rm=T))
df = df %>% mutate(T0_PS.CASSS_MEAN =
rowMeans(across(T0_PS.CASSS_1:T0_PS.CASSS_9), na.rm=T))
df = df %>% mutate(T0_CE_MEAN = rowMeans(across(T0_CE_1:T0_CE_10),
na.rm=T))
df = df %>% mutate(T0_TW_MEAN = rowMeans(across(T0_TW_1:T0_TW_7),
na.rm=T))
df = df %>% mutate(T0_TE_MEAN = rowMeans(across(T0_TE_1:T0_TE_6),
na.rm=T))

df = df %>% mutate(T2_WEMWBS_MEAN =
rowMeans(across(T2_WEMWBS_1:T2_WEMWBS_7), na.rm=T))
df = df %>% mutate(T2_PS.CASSS_MEAN =
rowMeans(across(T2_PS.CASSS_1:T2_PS.CASSS_9), na.rm=T))
df = df %>% mutate(T2_CE_MEAN = rowMeans(across(T2_CE_1:T2_CE_10),
na.rm=T))
df = df %>% mutate(T2_TW_MEAN = rowMeans(across(T2_TW_1:T2_TW_7),
na.rm=T))
df = df %>% mutate(T2_TE_MEAN = rowMeans(across(T2_TE_1:T2_TE_6),
na.rm=T))

df = df %>% mutate(T5_WEMWBS_MEAN =
rowMeans(across(T5_WEMWBS_1:T5_WEMWBS_7), na.rm=T))
df = df %>% mutate(T5_PS.CASSS_MEAN =
rowMeans(across(T5_PS.CASSS_1:T5_PS.CASSS_9), na.rm=T))
df = df %>% mutate(T5_CE_MEAN = rowMeans(across(T5_CE_1:T5_CE_10),
na.rm=T))
df = df %>% mutate(T5_TW_MEAN = rowMeans(across(T5_TW_1:T5_TW_7),
na.rm=T))
df = df %>% mutate(T5_TE_MEAN = rowMeans(across(T5_TE_1:T5_TE_6),
na.rm=T))

#put means before raw data (easy of tableby use)
df = df %>% relocate(T0_WEMWBS_MEAN:T5_TE_MEAN, .after =
Intervention)
```

```
#How many complete cases?
CountCompCases(df[1:19])

#Descriptive stats----
#produce descriptives table (by intervention)
descriptives_table = tableby(Intervention ~ ., data=df[2:39])
descriptives_by_intervention =
as.data.frame(summary(descriptives_table,
                                                    title =
"Sandwell"))

#Histograms
multi.hist(df[,c(5:39)])

#Reliability/consistency####

#Cronbach's alphas
Alphas_WEM_T0 = alpha(df[,5:11], na.rm = T)
Alphas_PS_T0 = alpha(df[,12:20], na.rm = T)
Alphas_CE_T0 = alpha(df[,21:30], na.rm = T)
Alphas_TW_T0 = alpha(df[,31:37], na.rm = T)
Alphas_TE_T0 = alpha(df[,38:43], na.rm = T)

Alphas_WEM_T2 = alpha(df[,83:89], na.rm = T)
Alphas_PS_T2 = alpha(df[,90:98], na.rm = T)
Alphas_CE_T2 = alpha(df[,99:108], na.rm = T)
Alphas_TW_T2 = alpha(df[,109:115], na.rm = T)
Alphas_TE_T2 = alpha(df[,77:83], na.rm = T)

Alphas_WEM_T5 = alpha(df[,171:178], na.rm = T)
Alphas_PS_T5 = alpha(df[,179:188], na.rm = T)
Alphas_CE_T5 = alpha(df[,189:199], na.rm = T)
Alphas_TW_T5 = alpha(df[,200:207], na.rm = T)
Alphas_TE_T5 = alpha(df[,208:214], na.rm = T)

#Model 1 - Comparing I/C scores for TW/TE----

#load long dataset
long = read.csv("long_T025.csv", na.strings = c("","NA"))
long %>% mutate_if(is.numeric,as.integer)

#LOO for groupings####
Q1_NULL_1 = brm(EMW ~ 1 + (Time|s|ID:School) + (Time|p|School),
              data = long,
              seed = 1234,
              chains = 4,
              iter = 5000,
              cores = 6)
Q1_NULL_1 = add_criterion(Q1_NULL_1, "loo")

Q1_NULL_1_IMP = brm_multiple(EMW ~ 1 + (Time|s|ID:School) + (Time|p|
School),
                data = imp_logged,
                seed = 1234,
```

```
                chains = 4,
                iter = 5000,
                cores = 6)
Q1_NULL_1_IMP = add_criterion(Q1_NULL_1_IMP, "loo", moment_match =
T)

Q1_NULL_2 = brm(EMW ~ Time + (Time|s|ID:School) + (Time|p|School),
                data = long,
                seed = 1234,
                chains = 4,
                iter = 5000,
                cores = 6)
Q1_NULL_2 = add_criterion(Q1_NULL_2, "loo")

Q1_NULL_3 = brm(EMW ~ Time + Intervention + (Time|s|ID:School) +
(Time|p|School),
                data = long,
                seed = 1234,
                chains = 4,
                iter = 5000,
                cores = 6)
Q1_NULL_3 = add_criterion(Q1_NULL_3, "loo")

Q1_NULL_4 = brm(EMW ~ Time + Intervention + (PS + CE + TW + TE)
                + (Time|s|ID:School) + (Time|p|School),
                data = long,
                seed = 1234,
                chains = 4,
                iter = 5000,
                cores = 6)
Q1_NULL_4 = add_criterion(Q1_NULL_4, "loo")

loo_compare(Q1_NULL_1, Q1_NULL_1_IMP, Q1_NULL_2, Q1_NULL_3,
Q1_NULL_4)

#LOO for time?####
mainmod_student_linear = brm(EMW ~ Time * Intervention * (PS + CE +
TW + TE)
                             + (Time|s|ID:School) + (Time|p|School),
                             family = student(),
                             data = long,
                             seed = 1234,
                             chains = 4,
                             iter = 10000,
                             cores = 6)
mainmod_student_linear = add_criterion(mainmod_student_linear,
"loo")
pp_check(mainmod_student_linear)

mainmod_student_poly_1 = brm(EMW ~ poly(Time,1) * Intervention * (PS
+ CE + TW + TE)
                             + (poly(Time,1)|s|ID:School) +
(poly(Time,1)|p|School),
                             family = student(),
```

```
                             data = long,
                             seed = 1234,
                             chains = 4,
                             iter = 10000,
                             cores = 6)
mainmod_student_poly_1 = add_criterion(mainmod_student_poly_1,
"loo")
pp_check(mainmod_student_poly_1)

#THIS IS THE MODEL
mainmod_student_poly2 = brm(EMW ~ poly(Time,2) * Intervention * (PS
+ CE + TW + TE)
              + (poly(Time,1)|s|ID:School) + (poly(Time,1)|p|
School),
              family = student(),
              data = long,
              seed = 1234,
              chains = 4,
              iter = 10000,
              cores = 6)
mainmod_student_poly2 = add_criterion(mainmod_student_poly2, "loo")
plot(mainmod_student_poly2)
pp_check(mainmod_student_poly2)
summary(mainmod_student_poly2)

#Check whether normal or student is most appropriate
loo_compare(mainmod_norm, mainmod_student_poly2)
#student_poly2 is -5.2 better, plus pp_check looks better

#Check how to model time
loo_compare(mainmod_student_poly2, mainmod_student_poly_1,
mainmod_student_linear)
#student_poly2 is better (linear = 0, poly1 = -1.2, poly2 = -9.0)

#What happens if we log transform
long_log = read.csv("long_T025.csv", na.strings = c("","NA"))
long_log[6:10] = log(long_log[6:10])

mainmod_student_poly2_logged = brm(EMW ~ poly(Time,2) * Intervention
* (PS + CE + TW + TE)
                            + (poly(Time,1)|s|ID:School) +
(poly(Time,1)|p|School),
                            family = student(),
                            data = long_log,
                            seed = 1234,
                            chains = 4,
                            iter = 10000,
                            cores = 6)
mainmod_student_poly2_logged =
add_criterion(mainmod_student_poly2_logged, "loo")
plot(mainmod_student_poly2_logged)
pp_check(mainmod_student_poly2_logged)
summary(mainmod_student_poly2_logged)
```

```
#compare the log-transformed model with the not log-transformed
model
loo_compare(mainmod_sratio, mainmod_student_poly2_logged)

#Log-transformed model is a LOT better (sratio=0, logged=-643.9)

mainmod_student_poly2_logged = brm(EMW ~ poly(Time,2) * Intervention
+ (poly(Time,1)|s|ID:School) + (poly(Time,1)|p|School),
                                   family = student(),
                                   data = long_log,
                                   seed = 1234,
                                   chains = 4,
                                   iter = 5000,
                                   cores = 6)
mainmod_student_poly2_logged =
add_criterion(mainmod_student_poly2_logged, "loo")
plot(mainmod_student_poly2_logged)
pp_check(mainmod_student_poly2_logged)
summary(mainmod_student_poly2_logged)


#lets now impute the missing data
imp_logged = mice(long_log, m = 5, print = FALSE)

mainmod_student_poly2_logged_imp = brm_multiple(EMW ~ poly(Time,2) *
Intervention * (PS + CE + TW + TE)
                                   + (poly(Time,1)|s|ID:School) +
(poly(Time,1)|p|School),
                                   family = student(),
                                   data = imp_logged,
                                   seed = 1234,
                                   chains = 4,
                                   iter = 5000,
                                   cores = 6)
mainmod_student_poly2_logged_imp =
add_criterion(mainmod_student_poly2_logged_imp, "loo")
plot(mainmod_student_poly2_logged_imp)
pp_check(mainmod_student_poly2_logged_imp)
summary(mainmod_student_poly2_logged_imp)

mainmod_student_poly2_logged = brm(EMW ~ poly(Time,2) * Intervention
* (PS + CE + TW + TE)
                                                 + (poly(Time,1)|s|
ID:School) + (poly(Time,1)|p|School),
                                                 family = student(),
                                                 data = long_log,
                                                 seed = 1234,
                                                 chains = 4,
                                                 iter = 5000,
                                                 cores = 6)
mainmod_student_poly2_logged =
add_criterion(mainmod_student_poly2_logged, "loo")
plot(mainmod_student_poly2_logged)
pp_check(mainmod_student_poly2_logged)
```

```
summary(mainmod_student_poly2_logged)

loo_compare(Q1_NULL_1_IMP, mainmod_student_poly2_logged_imp)
```
